# Supplementary material for: Learning from the past: A short term forecast method for the COVID-19 incidence curve
Source: PLoS Comput Biol. 2023 Jun 21;19(6):e1010790. doi: 10.1371/journal.pcbi.1010790 (PMC10317234; doi:10.1371/journal.pcbi.1010790)
Supplement: Attachment — Submitted filename: PLOS one reviews answers.pdf [file pcbi.1010790.s001.pdf]

Editor: Please pay special attention to Reviewer 1's comment about overall clarity and Reviewer 2's comments about review of related literature and the period of forecasting evaluation. These issues will need to be addressed compellingly for a positive outcome to be possible.

All comments by the reviewers copied below. Our answers in blue.

Comments to the Authors:

Please note here if the review is uploaded as an attachment.

Reviewer #1: This article deals with the covid-19 data for several countries (France, Germany, USA UK). Unfortunately, this article is not clearly written, so I can not give an opinion at this level.

From the abstract, their goal is to use some method called EpiInvertForecast, which is not explained in the paper

Authors: we have added a description of EpinInvert in Material and Methods. The forecast method itself is the object of the paper and is explained in the Introduction, detailed in Material and Methods, and its antecedents are discussed in the new paragraph of the Discussion entitled "The method of analogues". As stated in the "Material and Methods" section, in the EpiInvert R package, EpiLearn is executed using the EpiInvertForecast R function.

After (briefly) checking their explanation on the webpage dedicated to EpiInvertForecast, it looks like a series of standard statistical technics (I do not see anything new (possibly I miss understood it).

Authors: the forecast technique as it is used is not a standard statistical technique, although it can be related to kernel methods (for which we added references suggested by Reviewer#2).

In the "result" section, I do not understand where their results are.

Authors: The result section has been revised with more recent results.

At the moment, the paper is too confusing to say anything.

Authors: we have reduced and focused the Discussion section, we have detailed EpinInvert that is being used, and added context and references on the method of analogues, which is clearly an antecedent to our proposed forecast method.

Therefore I recommend significant modifications to clarify it.

Reviewer #2: Summary:

This article presents a method for forecasting infectious disease using a variation on the "method of analogues", and applies that modeling approach to forecasting COVID-19 incidence in Europe. In my view, this could eventually be publishable work

that applies a variation on an existing method to a novel setting. However, several points outlined in more detail below would need to be addressed before publication. Most importantly: (1) the literature review omits closely related work; (2) the evaluation time frame is far too short; and (3) a large section of the discussion could be omitted or much abbreviated. I don't see a satisfactory way to address the second of these points without either (a) waiting some time to gather more data on model performance, or (b) expanding the application to additional time frames and/or other locations [while taking care to make proper use of the versions of data that would have been available in real time].

Major comments:

- The introduction and literature review do not include some relevant literature. The method that is introduced in this paper is a variation on the "method of analogues", which has been applied to infectious disease forecasting in the past for both point forecasts and probabilistic forecasts; see the references in the points below. The authors should properly review the literature in the introduction and situate their methodological contributions appropriately. (To be clear: there are methodological innovations in the present work; I would just like to see the connection made to past similar work.)

(1) Viboud, Cécile, et al. "Prediction of the spread of influenza epidemics by the method of analogues." American Journal of Epidemiology 158.10 (2003): 996-1006.

(2) Ray, Evan L., et al. "Infectious disease prediction with kernel conditional density estimation." Statistics in medicine 36.30 (2017): 4908-4929.

Authors: we have revised thoroughly the literature review, added a discussion of the proposed references and more. This analysis is also reflected in the introduction where we link our proposed method to the "method of analogues" and to a recent attention based neural method.

- The evaluation time period of 2 months is extremely short. I would argue that this time frame is far too short to obtain reliable results about model performance. Evaluations of forecasts in similar settings have found that models can appear to do well for spans of several months at a time and then have substantial errors.

Authors: in the new version of the paper we have completely reformulated the presentation of the results. We now present the performance of forecast results from August, 6, 2022 to March 6, 2023 in Table 1. For example, in the case of a 1 week horizon we use 728 targets (each target corresponds to a combination of forecast date and country) to compute the method performance indicators. We believe that now, the time period and the number of targets is large enough to have an idea about the method performance.

- The level of detail in the discussion section seems out of place. It is valuable to include references to this previous work, but I would argue that this literature review could be shortened to three paragraphs or less with no impact on the communication of the main body of the work. For example, Table 1 could be omitted entirely; the parameter values that were selected for ARIMA models in 14 other published papers

have no bearing on the reader’s understanding or interpretation of the results for the methods described in this manuscript. Similarly, the commentary on compartmental models is not relevant or helpful to the main point of the current paper (and I’m not convinced by the authors’ arguments, as, e.g., depletion of susceptibles is a major driving force in the forecasts that are generated by these models, and is typically estimated by fitting the model to a time span of many weeks).

Authors: we have applied all of these recommendations. The discussion section has been reduced and refocused: we added to it a discussion of the proposed references (and more), including a discussion of papers using the method of analogues.

Additional substantive comments

On page 4, the authors write, “To add a curve of this type to the database, two conditions were imposed: the first was that the minimum time interval of the resulting sequence to apply EpiInvert was 150 days. The second condition was that the mean of the 56 values of the sequence must be larger than 1000. (Small averages can correspond to nonthreatening or neglected stages of the epidemic…)” I have a question about each of these conditions:

(1) I don’t understand the statement of the first condition; can the authors please clarify what is meant by “the minimum time interval of the resulting sequence to apply EpiInvert”?

(2) It would seem that eliminating reference curves with low incidence from the database would systematically bias the forecasts at times with low incidence. Can the authors comment on this?

Authors: We have completely reformulated the paragraph to clarify the questions raised by the reviewer. The new paragraph is copied below.

“Our proposed method, EpiLearn, uses a world-wide database of raw incidence curves from 61 countries and regions up to May 5, 2022. For each country or region, and for each day, starting 150 days after the beginning of the epidemic, we take the raw incidence data up to that day. Then, the resulting curve is further processed by applying the EpiInvert incidence decomposition algorithm \cite{AMM22Bio} (see the Material and Methods section) and we keep the last 56 values of the estimated incidence trend curve. To add a curve of this type to the database, we impose that the mean of the 56 values of the sequence must be larger than 1000. Taking into account that we normalize all database curves, the magnitude of the curves therefore has no influence in the forecast estimation. This amounts to making the assumption that the incidence curve evolution has the same behavior in large countries than in small countries. We impose this minimum 1000 cases average condition because for very small averages the registered incidence curves are often very noisy and unreliable. Indeed, small averages often correspond to non-threatening or neglected stages of the epidemic.”

- In Eq (4), it appears that the forecasts are scaled by the factor s_{28}/i^k_{28}, but in Equations (1) and (2) normalization was done so that the average of the first 28 days summed to 1. Can the authors comment on why two different scaling procedures were used? Would it not be better to pick one to use more consistently?

Authors: Equation (1) provides a normalization of the database and equation (2) provides a normalization of the current curve. In this way, the normalized current curve and the database curves can be compared. Once the most similar curves in the database to the normalized current curve are selected, we need to scale each database selected curve to fit the initial current one without normalization. The factor s_{28}/i^k_{28} is used to adjust each database curve to the current one before computing the median. This is now clarified in the text.

- Although forecasts are produced at horizons of 1 through 28 days ahead, the selection of the tuning parameters N_{median} and mu was done by examining only the errors at horizons of 1 through 14 days ahead. Why? Would different tuning parameters have been selected if evaluation was done based on the full forecast horizon that was used?

Authors: To clarify this point, we added the following paragraph to the new version of the paper.

"We optimized the parameters N_{median} and mu using the first 14 forecast days because the expected error in the next 14 days is so large that we prefer to focus on the optimization for the first 14 days. We could also optimize the above parameters for the whole 28 forecast days. In that case, we obtain as optimal values N_{median} = 128 and mu = 0.1075 which are slightly different from the ones obtained for the first 14 days."

- How well calibrated were the forecasts? Could the authors include, e.g., an evaluation of interval coverage rates or one-sided quantile coverage rates?

Authors: We have added in Table 1, the 50% and 95% interval coverage rates. These indicators are not quite representative of the quality of the solution. Indeed, they do not take into account how far the estimation is from the confidence intervals. The low value (comparing with other methods) of the relative weighted interval score (wis) indicates instead that, globally, the estimations obtained by EpiLearn are quite close to the confidence intervals.

- The method accounts for a trend (and uncertainty about the trend) and a weekly cycle in reporting. However, it seems that additional noise is not captured. As described in the discussion section, the EpiInvert method that is used for preprocessing decomposes the series into trend, seasonality, and noise components; the noise seems to be lost in subsequent processing in the proposed algorithm, though.

Authors: the noise model for the trend curve estimated by EpiInvert does not include any forecast procedure. In this paper, we do not formulate any model for the noise of the forecast of the trend curve (that could be far from trivial). We just study, empirically, the forecast confidence intervals.

- “Since EpiLearn forecasts the daily incidence, the weekly forecast is obtained by summing the forecasted raw daily incidence given by (5). The quantiles of the associated weekly distributions are computed on the registered database of incidence curves by extending the procedure of section which computes the confidence intervals of the forecasted incidence curve.”

(1) The procedure for obtaining weekly quantiles is not completely clear: do you aggregate to the weekly scale first and then compute quantiles, or do you compute quantiles on the daily scale and then aggregate? I think you are doing the second of these, but it would be helpful to be precise.

(2) If indeed you are calculating quantiles on a daily scale and then aggregating, I think that you are implicitly assuming perfect dependence in the forecast distributions across days. This assumption is not true, but this results in an inflation of uncertainty. Please comment on this.

Authors: We aggregate to the weekly scale first and then compute quantiles. This point is clarified in the new version of the paper.

- It would be helpful to include a figure showing the actual observed disease incidence during the time span evaluated in the section on Comparative results from the European COVID-19 Forecast Hub. Was this a time with “interesting” behavior in disease incidence trends?

Authors: following the reviewer's suggestions we added Figure 5 in the new version of the paper.

- As the authors discuss in the Materials and Methods section, infectious disease surveillance data are subject to revision. In evaluations of forecasting methods, it is critical to address this by using the version of the data that would have been available in real time when producing forecasts. It is my understanding that this was done, but a clear statement of this would be beneficial. (If it was not done, the analysis should be reworked.)

Authors: to evaluate the performance of the methods we only used the results provided by the European Hub. These results cannot be manipulated and they use the version of the data that would have been available in real time when producing forecasts. We clarify this point in the new version of the paper.

Minor comments:

- Around Eq (5), it would be helpful to define % as the modulus operator.

Authors: done

- The sentence “Fig 3 shows the function f (x) = e−0.0475x which determines the weight assigned to each day in the past in the distance estimation.” on page 7 would better belong just after Eq (3), where the exponential weighting is first described.

Authors: done

- There is some notational inconsistency in the use of the subscript m:

(1) In Eq (6), the authors define \hat{u}_m to denote a forecast at horizon m, but in Eq (7) and (9) a subscript of m+28 is used.

(2) Later, m is used yet again to index the forecast model

Authors: Now, we use the index j for the normalization step, the index d for the forecast day and the index m for the model.

- Typo near the top of page 8 where the subscript 0.0975 is used rather than 0.975

Authors: done

- Throughout the manuscript, section references are missing

Embarrassingly, PLOS computational does not allow for section numbering. We therefore referred to sections by their name, “Introduction”, “Results”, “Comparative Results”.

- There are minor spelling and grammatical issues throughout the manuscript that would benefit from the attention of an editor.

Have the authors made all data and (if applicable) computational code underlying the findings in their manuscript fully available?

The PLOS Data policy requires authors to make all data and code underlying the findings described in their manuscript fully available without restriction, with rare exception (please refer to the Data Availability Statement in the manuscript PDF file). The data and code should be provided as part of the manuscript or its supporting information, or deposited to a public repository. For example, in addition to summary statistics, the data points behind means, medians and variance measures should be available. If there are restrictions on publicly sharing data or code —e.g. participant privacy or use of data from a third party—those must be specified.

Reviewer #1: Yes

Reviewer #2: Yes

PLOS authors have the option to publish the peer review history of their article (what does this mean?). If published, this will include your full peer review and any attached files.

If you choose “no”, your identity will remain anonymous but your review may still be made public.

Do you want your identity to be public for this peer review? For information about this choice, including consent withdrawal, please see our Privacy Policy.

Reviewer #1: No

Reviewer #2: **Yes:** Evan L. Ray

Figure Files:

While revising your submission, please upload your figure files to the Preflight Analysis and Conversion Engine (PACE) digital diagnostic tool, https://pacev2.apexcovantage.com. PACE helps ensure that figures meet PLOS requirements. To use PACE, you must first register as a user. Then, login and navigate to the UPLOAD tab, where you will find detailed instructions on how to use the tool. If you encounter any issues or have any questions when using PACE, please email us at figures@plos.org.

Data Requirements:

Please note that, as a condition of publication, PLOS' data policy requires that you make available all data used to draw the conclusions outlined in your manuscript. Data must be deposited in an appropriate repository, included within the body of the manuscript, or uploaded as supporting information. This includes all numerical values that were used to generate graphs, histograms etc.. For an example in PLOS Biology see here: http://www.plosbiology.org/article/info%3Adoi%2F10.1371%2Fjournal.pbio.1001908#s5.

Reproducibility:

To enhance the reproducibility of your results, we recommend that you deposit your laboratory protocols in protocols.io, where a protocol can be assigned its own identifier (DOI) such that it can be cited independently in thedtui bcin future. Additionally, PLOS ONE offers an option to publish peer-reviewed clinical study protocols. Read more information on sharing protocols at https://plos.org/protocols?utm_medium=editorial-email&utm_source=authorletters&utm_campaign=protocols
