# Supplementary material for: Learning from the past: A short term forecast method for the COVID-19 incidence curve
Source: PLoS Comput Biol. 2023 Jun 21;19(6):e1010790. doi: 10.1371/journal.pcbi.1010790 (PMC10317234; doi:10.1371/journal.pcbi.1010790)
Supplement: Attachment — Submitted filename: PLOS rebuttal 2.pdf [file pcbi.1010790.s002.pdf]

Dear Prof. Morel,

Thank you very much for submitting your manuscript "Learning from the past: a short term forecast method for the COVID-19 incidence curve" for consideration at PLOS Computational Biology.

As with all papers reviewed by the journal, your manuscript was reviewed by members of the editorial board and by several independent reviewers. In light of the reviews (below this email), we would like to invite the resubmission of a significantly-revised version that takes into account the reviewers' comments.

We cannot make any decision about publication until we have seen the revised manuscript and your response to the reviewers' comments. Your revised manuscript is also likely to be sent to reviewers for further evaluation.

When you are ready to resubmit, please upload the following:

[1] A letter containing a detailed list of your responses to the review comments and a description of the changes you have made in the manuscript. Please note while forming your response, if your article is accepted, you may have the opportunity to make the peer review history publicly available. The record will include editor decision letters (with reviews) and your responses to reviewer comments. If eligible, we will contact you to opt in or out.

[2] Two versions of the revised manuscript: one with either highlights or tracked changes denoting where the text has been changed; the other a clean version (uploaded as the manuscript file).

Important additional instructions are given below your reviewer comments.

Please prepare and submit your revised manuscript within 60 days. If you anticipate any delay, please let us know the expected resubmission date by replying to this email. Please note that revised manuscripts received after the 60-day due date may require evaluation and peer review similar to newly submitted manuscripts.

Thank you again for your submission. We hope that our editorial process has been constructive so far, and we welcome your feedback at any time. Please don't hesitate to contact us if you have any questions or comments.

Sincerely,

Alex Perkins
Academic Editor
PLOS Computational Biology

Lucy Houghton
Staff
PLOS Computational Biology

****

Reviewer's Responses to Questions

Comments to the Authors:
Please note here if the review is uploaded as an attachment.

Reviewer #2: Summary:
I continue to believe that the work described in this manuscript should be published; it describes a successful application of a method with demonstrated strong performance relative to other methods for forecasting COVID-19 incidence. The majority of my comments on the first submission have been successfully addressed in this revision, but I still have a number of relatively minor comments remaining, which primarily relate to the readability and clarity of the paper rather than substantive comments on the methods or evaluation. That said, I am suggesting another round of “major revisions” due to the number of comments here.

Comments on methods:
1. Although nearly all of the points I raised in the initial submission were addressed, there is one I would like to follow up on. I reproduce the discussion here.

My initial comment was “The method accounts for a trend (and uncertainty about the trend) and a weekly cycle in reporting. However, it seems that additional noise is not captured. As described in the discussion section, the EpiInvert method that is used for preprocessing decomposes the series into trend, seasonality, and noise components; the noise seems to be lost in subsequent processing in the proposed algorithm, though.”

The authors’ response was: “the noise model for the trend curve estimated by EpiInvert does not include any forecast procedure. In this paper, we do not formulate any model for the noise of the forecast of the trend curve (that could be far from trivial). We just study, empirically, the forecast confidence intervals.”

My follow-up comment: At minimum, it seems worth explicitly acknowledging the fact that noise around the trend is not modeled as a limitation of the method. It seems possible that this might be a contributing factor to the lower-than-nominal interval coverage rates reported in the updated Table 1. It would also be nice to include any ideas you have for possible approaches to addressing this in the discussion for future work. For example, might it be possible to extract residuals and model their distribution on a per-location basis?

Author's response: First of all, thank you very much for this second careful reading and suggestions.

We use the EpiInvert method, which performs a decomposition of the signal into trend and noise after correction of the weekly bias. In the present paper we only use the trend curves estimated by EpiInvert, because what is forecast also is a trend. Thus, it is a “trend to trend” prediction. Thus, you are absolutely right to point out that we did not predict the additional error between prediction and ground truth that is caused by the noise component. Our error prediction is only concerned about the method’s bias, namely the observed inherent variability of the future trends following a given trend interval. Thus, a further refinement of

the method should be to keep the residual furnished by EpinInvert for a given incidence curve, estimate its model, and deduce a noise variance for the prediction. This noise variance should be added to the method bias variance. We added this remark in our comments on Table 1 in the following form:

“The EpiInvert method performs a decomposition of the past incidence curve into a trend and a noise component, after correction of the weekly bias. In the above error estimations, we only used the trend curves, because what is forecast also is a trend. We did not compute the additional error between prediction and ground truth that is caused by the noise component. Our above error prediction therefore only addresses the method’s bias, namely the observed variability of the future trends following a given past trend interval. A further refinement of the method should take into account the noise residual computed by EpiInvert for a given incidence curve, estimate its model, and deduce a noise variance for the prediction. This noise variance should be added to the method bias variance..”

2. In the paragraph describing the Data preprocessing method, there is a comment that the serial interval is observable. What is meant by this? It is my impression that most public health surveillance data sets do not have sufficient resolution to observe the serial interval, since we do not have information about secondary cases. Are you simply using estimates of the serial interval reported in the cited reference, Ma et al.? If so, it would be helpful to state this and the specific value used.

Author's response: Yes, we use Ma est al.’s serial interval, which is not just a specific value but a probability distribution that was evaluated on about 1000 verified transmission pairs at the beginning of the Covid 19 pandemic. By observable we meant that in opposition to the infection time, the time at which symptoms appear is observable. In any case, to avoid misinterpretation, we have removed the word “observable”. We clarified this in the text:

Data preprocessing method The infectiousness of individuals at time t is characterized by the reproduction number R_t, defined as the average number of cases generated by an infected person at time t, and by the (observable) serial interval Φ_s which represents the time distribution of the delay of the onset of symptoms between primary and secondary cases. For Covid 19, this serial interval was measured accurately in [12] on nearly 1000 verified transmission pairs. We use this distribution in the EpiInvert method.

3. Confidence intervals are currently based on a global distribution of relative errors. However, I might imagine that the size of relative errors might vary depending on the epidemic phase. It might be interesting to examine this with a plot similar to Fig 4 examining the distribution of relative errors as obtained for example in phases of steady low disease incidence, increasing incidence, high incidence, and low incidence. The motivating question here is whether it might make sense to calculate prediction intervals based on quantiles of the selected reference trajectories from the database rather than global relative errors?

Author's response:

It might indeed be relevant to make error statistics taking into account the shape of the query curve, and the low incidence or high incidence as well. Currently the size of the

incidence is not taken into account because the query curves are normalized. Using unnormalized curves would reduce drastically the number of similar samples, which is the reason why we did not adopt this strategy. A future refinement of the method could be to add an additional term to the computed distance between curves (equation (6)), representing the overall incidence over the period of time, normalized to the population of the country. The forecasting could also benefit from additional knowledge about changes in social distancing policy and evolution of the virus' contagiousness (as was observed with the emergence of Omicron). However, when designing the method, we opted to make it as generally applicable as possible and avoid case-by-case adjustments. Digging into these aspects requires a far ranging overhaul of our experimental protocol which belongs to our future plans. We added adequate comments including your suggestion in the Discussion section.

Paragraph added to the conclusion: *The proposed method might be improved in several ways by taking into account additional relevant factors before comparing time sequences. Indeed, our obtained confidence intervals were based on a global distribution of relative errors. However, the size of relative errors might vary depending on the trend and magnitude of the query curve. It might be interesting to explore this by adding a distance of the average incidences as an additional term in the distance between incidences given in equation (6). In addition, the forecasting could benefit from additional knowledge about implementation or changes of the social distancing policy and evolution of the virus' contagiousness (as was observed with the emergence of Omicron). Digging into these aspects requires a far ranging overhaul of our experimental protocol which belongs to our future plans.*

Comments on organization:

4. I suggest moving the contents of the "discussion" section up to the introduction. This material is really a literature review, and so I think it would be more standard to place it near the beginning of the article.

Author's response: We have moved the method of analogues to the introduction. The analysis of the other methods has been kept at the end, because it is interlaced with the discussion and refers repeatedly to the main Figures 5 and 6 in Results.

5. I suggest that the first several subsections of the results would be better placed under Materials and Methods, including "Incidence trend curves database construction using EpiInvert", "Normalization of the database incidence curves", "Computing the distance between curves", "Forecasting using a median of the closest database curves", "Choice of the method parameters", and "Empirical confidence intervals." The sections describe aspects of the methodology rather than results.

Author's response: Done: we have moved down the Results section.

Comments on notation:
6. In the section on "Choice of method parameters", it seems like there is some inconsistency in notation around equations (9) and (10). The second sentence reads "Set, for each i^k in the database, …", but i^k does not appear in Eq (9). Do you mean for each

s^k in the database? In the following sentence you have s^k without a hat, and Eq (10) refers to i^k_{d+28} which I think should correspond to \hat{s}^k_{d+28} in the notation of Eq (9) or s^k in the text just after Eq (9). I have similar questions about the notation in the following section on “Empirical confidence intervals”: is s^k being used here to refer to the first 28 entries of i^k? Could we then just pick one letter, either using i^k with appropriate indexes or s^k and s^f? Am noting that the lack of a k index on s^f is also confusing. Summing up: I think it would be helpful to establish early on a consistent notation for a curve that is being forecasted and a reference curve from the database, and use this standardized notation throughout.

Author's response: you are right. The notation was confusing. In the new version of the paper, at the beginning of the method presentation we have introduced consistent notation to manage the incidence curves and their forecast and we have corrected the manuscript text accordingly. Changes are in blue.

7. Similarly, it seems like it might be possible to establish a single notation for a relative forecast error to use throughout the sections on choice of method parameter and empirical confidence intervals. We currently have e^d,k and e_d,k which differ only in whether the numerator involves an absolute value and whether the error is regarded as a function of the model hyperparameters N_{median} and mu. Could this discussion be streamlined in terms of notation, for example using only the e_d,k notation that is currently introduced in Eq (12) currently, and in Eq (11) summing the absolute errors and discussing the dependence of the hyperparameters in the text?

Author's response: Indeed the definition of e^d,k was unnecessary duplicated, we have rewritten the concerning paragraph to address this issue. Changes are in blue.

8. It is not clear why in Eq (9) the numerator involves \hat{s}^k_28, in contrast to the numerator of s_28 (without a hat) that is used when generating forecasts in Eq (7). My first thought was that this was done to remove scale effects in the unnormalized s, but that should be unnecessary since the relative error metric in Eq (10) should account for this already. Could we then simplify the presentation and notation by re-using the notation of Eq (7) for a forecast rather than introducing the new notation \hat{u}^k_d?

Author's response: In fact, as the database curves are normalized, \hat{s}^k = s^k. But as suggested by the reviewer it is better to keep the notation used in (7). We fully agree that the notation \hat{u}^k_d is not necessary, we have removed this notation and used instead an extension of notation of (7). Changes are in blue.

9. In Eq (6), when computing the similarity of the normalized vectors \hat{s} and i^k, should the comparison be based on i^k_j, i.e., we compare entry j of \hat{s} to entry j of i^k? The subscript p seems out of place.

Author's response: you are absolutely right again, p was out of place. We have corrected the equation using the appropriate subscript.

Misc. minor comments on word choice, etc:
10. In introduction: “the methods that were publicly available…” -> capitalize “The”

Author's response: Corrected, thank you.

11. “Our learning technique is widely different from previous methods introduced in the literature.” The revised manuscript now includes a review of a few similar papers, including one paper that was applied to forecasting COVID-19. Maybe revise the statement here to something that is a little more qualified, e.g. “Our learning technique is different in structure from most previous methods introduced in the literature.”

Author's response: Changed as suggested.

12. ”A weekly and festive bias corrected incidence …” Unclear what is meant by “festive” here; suggest another word choice.
Author's response: Done, we removed the mention of a correction of festive days, which is a further refinement of the method that is irrelevant here.

13. In the section on “Forecasting using a median of the closest database curves”, change “criterium” to “criterion”.

Author's response: Corrected, thank you.

14. There is text that did not render properly at the bottom of the section on “Choice of method parameters”

Author's response: Corrected, thank you.

15. Fig 6 is somewhat confusing because the bars for relative WIS are not shown for the ILM-EKF method; this makes it appear that the method is quite good, but I think that actually its performance is “off the charts” in terms of poor performance and the bar was not displayed.

Author's response: These values were above 10, and didn’t appear because they were out of axis bounds. We corrected the figure. Thank you!

16. In the discussion, ensemble methods are described as follows: “At every step, the ensemble fits a new learner to the difference between the observed response and the aggregated prediction of all learners grown previously.” This appears to be a description of boosting methods, which is one of many approaches to ensembling, and is not the method used in references 17 and 20. I suggest removing this sentence, as well as the sentence immediately after it.

Author's response: Done, thank you.

17. I appreciate the addition of the new Figure 5; this provides valuable context. The font size is quite small, though. It might be helpful to make the figure slightly larger or show data only for selected locations (perhaps with all locations shown in a supplemental figure), to allow for a larger font size.

Author's response: Done, thank you.

Have the authors made all data and (if applicable) computational code underlying the findings in their manuscript fully available?
The PLOS Data policy requires authors to make all data and code underlying the findings described in their manuscript fully available without restriction, with rare exception (please refer to the Data Availability Statement in the manuscript PDF file). The data and code should be provided as part of the manuscript or its supporting information, or deposited to a public repository. For example, in addition to summary statistics, the data points behind means, medians and variance measures should be available. If there are restrictions on publicly sharing data or code —e.g. participant privacy or use of data from a third party—those must be specified.

Reviewer #2: Yes

PLOS authors have the option to publish the peer review history of their article (what does this mean?). If published, this will include your full peer review and any attached files.

If you choose “no”, your identity will remain anonymous but your review may still be made public.

Do you want your identity to be public for this peer review? For information about this choice, including consent withdrawal, please see our Privacy Policy.

Reviewer #2: **Yes:** Evan L. Ray

Figure Files:

While revising your submission, please upload your figure files to the Preflight Analysis and Conversion Engine (PACE) digital diagnostic tool, https://pacev2.apexcovantage.com. PACE helps ensure that figures meet PLOS requirements. To use PACE, you must first register as a user. Then, login and navigate to the UPLOAD tab, where you will find detailed instructions

on how to use the tool. If you encounter any issues or have any questions when using PACE, please email us at figures@plos.org.
